# *Chlamydomonas* DYX1C1/PF23 is essential for axonemal assembly and proper morphology of inner dynein arms

**DOI:** 10.1371/journal.pgen.1006996

**Published:** 2017-09-11

**Authors:** Ryosuke Yamamoto, Jagan M. Obbineni, Lea M. Alford, Takahiro Ide, Mikito Owa, Juyeon Hwang, Takahide Kon, Kazuo Inaba, Noliyanda James, Stephen M. King, Takashi Ishikawa, Winfield S. Sale, Susan K. Dutcher

**Affiliations:** 1 Department of Biological Sciences, Graduate School of Science, Osaka University, Osaka, Japan; 2 Laboratory of Biomolecular Research, Paul Scherrer Institute, Villigen PSI, Switzerland; 3 Department of Biology, Oglethorpe University, Atlanta, Georgia, United States of America; 4 Department of Biological Sciences, Graduate School of Science, University of Tokyo, Tokyo, Japan; 5 Department of Cell Biology, Emory University School of Medicine, Atlanta, Georgia, United States of America; 6 Shimoda Marine Research Center, University of Tsukuba, Shizuoka, Japan; 7 Department of Genetics, Washington University School of Medicine, St. Louis, Missouri, United States of America; 8 Department of Molecular Biology and Biophysics, University of Connecticut Health Center, Farmington, Connecticut, United States of America; Stanford University School of Medicine, UNITED STATES

## Abstract

Cytoplasmic assembly of ciliary dyneins, a process known as preassembly, requires numerous non-dynein proteins, but the identities and functions of these proteins are not fully elucidated. Here, we show that the classical *Chlamydomonas* motility mutant *pf23* is defective in the *Chlamydomonas* homolog of *DYX1C1*. The *pf23* mutant has a 494 bp deletion in the *DYX1C1* gene and expresses a shorter DYX1C1 protein in the cytoplasm. Structural analyses, using cryo-ET, reveal that *pf23* axonemes lack most of the inner dynein arms. Spectral counting confirms that DYX1C1 is essential for the assembly of the majority of ciliary inner dynein arms (IDA) as well as a fraction of the outer dynein arms (ODA). A C-terminal truncation of DYX1C1 shows a reduction in a subset of these ciliary IDAs. Sucrose gradients of cytoplasmic extracts show that preassembled ciliary dyneins are reduced compared to wild-type, which suggests an important role in dynein complex stability. The role of PF23/DYX1C1 remains unknown, but we suggest that DYX1C1 could provide a scaffold for macromolecular assembly.

## Author summary

Most animal cells have antenna-like organelles called “cilia”. These organelles have various important functions both in motility and sensing the environment. Motile cilia are essential for moving cells as well as moving fluids across a surface. The waveform of motile cilia requires large macromolecular motors; these are the ciliary dyneins. These dynein complexes are assembled in the cytoplasm in a pathway called preassembly, and then transported into cilia. Defects in this process cause a heterogeneous human disease called primary ciliary dyskinesia that results, for example, in the disruption of the motility of respiratory tract cilia, sperm and nodal cilia during development. The mechanisms of the preassembly pathway are not fully understood. In this study, we use a mutation in the well-conserved *DYX1C1/PF23* gene of the green alga, *Chlamydomonas reinhardtii*. Loss of a conserved domain (DYX) reveals a failure to assemble most ciliary dyneins. Preassembly of inner arm dyneins is particularly affected. We find that if dynein arms are not assembled, dynein subunits in the cytoplasm are unstable. We suggest that DYX1C1 may play a role as a scaffold for other preassembly factors and the dynein subunits.

## Introduction

Motile cilia (also known as flagella) are antenna-like organelles protruding from many types of cells and required for motility and cell signaling [1, 2]. Motile cilia are essential for normal vertebrate development, fertility, and organ homeostasis [3–7]. The movement of motile cilia is driven by the ciliary dynein motors, which are composed of outer (ODA) and inner dynein arms (IDA) [8–10]. ODA and IDA motors are composed of different proteins and have different structures. The ciliary dyneins are assembled in the cytoplasm, a process called “preassembly”, before they are transported into cilia for docking on axonemal doublet microtubules [11, 12]. Defects in preassembly, transport or docking of the dynein complexes can cause abnormal ciliary motility and disorders in humans including primary cilia dyskinesia (PCD) [6, 13, 14]. Multiple non-dynein cytoplasmic proteins have been identified that are required for preassembly of the axonemal dyneins (reviewed in [15, 16]). These include ODA7/LRRC50/DNAAF1, PF13/KTU/DNAAF2, PF22/DNAAF3, HEATR2, IDA10/MOT48, ODA16, ZYMD10, PIH1D3 and LRRC6 [17–24] (see S1 Table). However, neither the molecular mechanism of the preassembly process, nor the complete genetic/functional relationship between these factors is known.

Dyslexia is a disorder in which the patients show normal intelligence but have problems in reading, writing and/or spelling words. The causative genes for dyslexia are controversial [25–32]. One candidate gene is *DYX1C1* (dyslexia susceptibility 1 candidate 1), which was disrupted by a translocation in dyslexia patients [33], but there is little validation of the role of this gene [34]. Recently, the DYX1C1 gene has also been implicated in ciliary assembly [35, 36], but these patients did not show dyslexia [35]. Chandrasekar et al [36] found that knockdown of the DYX1C1 homolog in zebrafish shows ciliary phenotypes that included a failure in assembly of some ciliary dyneins and abnormal ciliary motility. DYX1C1 mutations in human cause PCD due to abnormal ciliary motility and failure in assembly of axonemal dyneins [35]. The authors postulated that DYX1C1 (also called DNAAF4) is required for preassembly of ciliary dyneins, possibly working in concert with PF13/KTU/DNAAF2 [35]. However, the mechanism by which DYX1C1 affects dynein preassembly remained to be fully elucidated.

In this study, we report that the *Chlamydomonas* homolog of DYX1C1 is defective in the classical *Chlamydomonas* mutant *pf23* (*paralyzed flagella 23*), a paralyzed mutant deficient in ciliary dynein assembly [37]. *pf23* has been widely used to study the function and composition of ciliary dyneins (e.g. [38–42]). In *pf23*, DYX1C1 contains a mutation resulting in deletion of 27 amino acids within the DYX domain. The *pf23* phenotype is rescued by transformation with the wild-type *DYX1C1* gene, which indicates the deletion mutation in *PF23*/*DYX1C1* is sufficient to cause ciliary dynein assembly defects and the non-motile phenotype. Furthermore, cryo-electron-microscopic tomography (cryo-ET) and biochemical analyses reveal that the defects in axonemal dynein assembly are more profound than had previously been appreciated [37, 39, 40]. PF23/DYX1C1 is localized to the cytoplasm and is required for preassembly of most axonemal dyneins. In addition, missing dyneins in *pf23* display a range of defects in their subunit composition, and suggest PF23/DYX1C1 is essential for proper stability of axonemal dynein heavy chains. Thus, PF23/DYX1C1 is a conserved, cytoplasmic ciliary dynein assembly protein.

## Results and discussion

### *Chlamydomonas* has a single DYX1C1 gene

**Fig 1:**
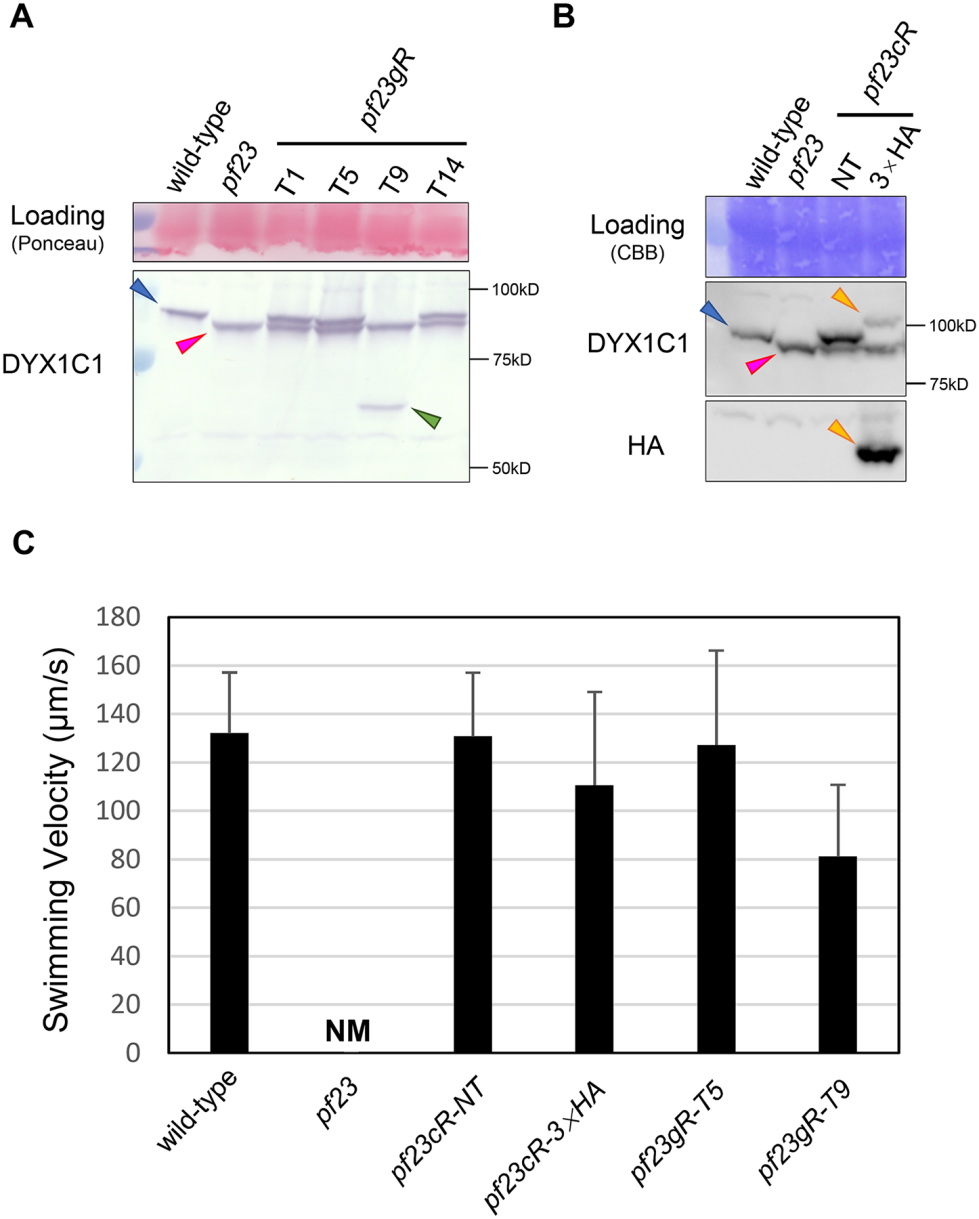
Immunoblot analyses reveal DYX1C1 is shorter in *pf23* and the wild-type *DYX1C1* gene rescues the *pf23* mutant. (A) Immunoblotting of whole cell samples from wild-type, *pf23*, *pf23gR* (T1, T5, T9, T14) using the anti-DYX1C1 antibody. Wild-type cells express an ~95 kDa DYX1C1 (blue arrowhead), while *pf23* expresses an ~92 kDa DYX1C1 (pink arrowhead), because of the loss of 27 amino acids that correspond to the wild-type 5^th^ exon. Rescued strains (T1, T5 and T14) expressed both wild-type and mutant-type DYX1C1. Note that *pf23gR-T9* expresses a short and reduced, but partially functional ~67 kDa DYX1C1 (green arrowhead, see text and Fig 3). (B) Immunoblotting of whole cell samples from wild-type, *pf23*, *pf23cR* (NT and 3×HA) using the anti-DYX1C1 and anti-HA antibodies. *pf23cR-NT* strains expressed both wild-type and mutant DYX1C1. *pf23cR-3×HA* strain expressed mutant and wild-type DYX1C1 tagged with 3×HA epitopes (orange arrowhead). This DYX1C1-3×HA in *pf23cR-3×HA* was also detected by the anti-HA antibody (orange arrowhead). The membrane, blotted with the DYX1C1 antibody (middle), was re-blotted with the anti-HA antibody (bottom). (C) Restoration of swimming in rescued *pf23* strains. Swimming velocity was measured for wild-type and rescued strains of *pf23*. 20–25 cells were selected for measurement of swimming speed. The original *pf23* is completely non-motile, and for the *pf23* strain “NM” indicates non-motile/not measured. Note that *pf23gR-T9* shows slower swimming compared to wild-type, possibly because of the reduced expression of the truncated DYX1C1 fragment and the resultant partial failure of inner dynein arm assembly (discussed in the text).

To determine the function of DYX1C1 in cilia-related processes, we searched the *Chlamydomonas* genome database (version 4 (http://genome.jgi.doe.gov/Chlre4/Chlre4.home.html) and version 5 (https://phytozome.jgi.doe.gov/pz/portal.html%23!info?alias=Org_Creinhardtii)) for DYX1C1 homologs. BLAST search using human DYX1C1 protein as the query revealed one copy of a DYX1C1 homolog in *Chlamydomonas* (Cre11.g467560 at Phytozome *Chlamydomonas* v5.5). The predicted *DYX1C1* sequences in both genome databases contained gaps, and the correct *Chlamydomonas DYX1C1* sequence was determined by PCR (the sequenced *Chlamydomonas DYX1C1* sequence has been deposited in the DNA Data Bank of Japan (DDBJ) under the accession number LC149873). The *Chlamydomonas DYX1C1* gene maps to chromosome XI near the *PF23* locus and is predicted to encode a protein with 816 amino acids and a theoretical pI/MW of 5.04/82751.23 (S1A and S1B Fig). The *Chlamydomonas* DYX1C1 protein is longer, with a lower pI, compared to the human DYX1C1 protein, which has 420 amino acids and a theoretical pI/Mw of 8.88/48526.88 (Figs 1A, S1A and S1B).

A search of *Chlamydomonas* DYX1C1, using the SMART (http://smart.embl-heidelberg.de/) and pfam (http://pfam.xfam.org/) servers, revealed a CS domain in the N-terminus; CS domains are predicted to be an interacting module for HSP90 (discussed further below; [18, 43]). The analysis also reveals a central coiled-coil domain and multiple TPR (tetratricopeptide repeat) motifs in the C-terminal half of the molecule, which are involved in protein-protein interactions (S1A Fig). Although there are some differences in the number of TPR motifs, the overall DYX1C1 domain structure is conserved in ciliated organisms that include human, mouse, zebrafish, *Trypanosoma* and *Chlamydomonas* (S1A and S1B Fig). This domain organization suggests that DYX1C1 is designed for protein interactions [35, 36].

### The *Chlamydomonas* mutant *pf23* is defective in the *DYX1C1* gene

Cross sections of ciliary axonemes in DYX1C1 morpholino knockdown zebrafish and the PCD patients reveal a partial failure of assembly of dynein arms [35, 36]. This structural phenotype is reminiscent of the axonemal phenotype of the *Chlamydomonas pf23* mutant, which lacks several inner dynein arms [37]. Therefore, we sequenced *DYX1C1* in *pf23*. Analysis of the *DYX1C1* genomic DNA in *pf23* reveals a 494 bp deletion that removes all of exon 5 and part of the flanking introns. Using a marker designed for this deletion and crosses between *pf23* and a mapping strain (S1D2: CC-2290), we confirmed that this deletion cosegregates with the Pf23 phenotype (62/62). The *pf23 DYX1C1* cDNA lacks 81 nucleotides of exon 5. The mutation results in the removal of 27 amino acids in DYX1C1, which forms part of the “DYX domain” [36] (Figs 1A and S1A). In addition, cDNA sequencing revealed that in *pf23* DYX1C1 exons 4 and 6 are directly connected in-frame (S1A Fig). Consistent with the sequencing data, immunoblots, using anti-*Chlamydomonas* DYX1C1 antibody (CT299; see Materials and methods), revealed that *pf23* expresses an altered, non-functional form of DYX1C1 (~ 92 kDa) while wild-type expresses a ~ 95 kDa form (Figs 1A, 1B, S2A and S2B).

To further test whether the defective DYX1C1 gene is responsible for the non-motile phenotype, we transformed *pf23* with the wild-type *DYX1C1* genomic DNA with its endogenous promoter or cDNA with the PsaD promoter. Both the wild-type *DYX1C1* genomic DNA and cDNA successfully rescued the *pf23* phenotype. The rescued strains (listed in S2 Table) show nearly wild-type motility (Fig 1C). Consistently, immunoblots of whole cells from the rescued strains show both the wild-type DYX1C1 together with the shorter, mutant DYX1C1 (Fig 1B). Also, the ciliary lengths of the rescued strains are near wild-type in the SG (Sager and Granick) media (wild-type, 10.9 ± 1.1 μm; *pf23*, 4.8 ± 0.6 μm; *pf23cR-NT*, 11.0 ± 1.2 μm, *pf23cR-3×HA*, 11.1 ± 2.0 μm; *pf23gR-T5*, 11.6 ± 1.4 μm; *pf23gR-T9*, 10.3 ± 1.4 μm; n = 10, see also [44]). These results strongly suggest that DYX1C1 is the gene responsible for the *pf23* phenotype, and that an intact DYX domain is essential for ciliary dynein assembly in *pf23* [37].

One transformant, *pf23gR-T9* (S2 Table), displays slightly slower swimming speeds compared to wild-type or other transformants (Fig 1C). In addition, immunoblots revealed a DYX1C1 fragment of ~ 67 kDa in cells from *pf23gR-T9* (Fig 1A). Sequence analysis of the inserted, exogenous DYX1C1 sequence in *pf23gR-T9* revealed that during transformation the full-length *DYX1C1* gene was not integrated, which predicts the loss of 208 amino acids at the C-terminus of the DYX1C1 protein (S1A Fig). The predicted pI/Mw of exogenous DYX1C1 in *pf23gR-T9* was 5.22/62780.39, consistent with the last two TPR motifs of DYX1C1 being lost and/or disrupted (Figs 1A and S2B). Conclusions are confounded by the observation that the DYX1C1 protein in *pf23gR-T9* is present at a lower level. These results may indicate that while the DYX domain is required for function, either the C-terminal two TPR motifs are required for the assembly of only specific inner dynein arms, or inner dynein arm assembly is affected more by the amount of DXY1C1, or both (Figs 1A and S1A). This C-terminal truncation would produce a protein that is similar to that encoded by the translocation found in the Finnish family with dyslexia [33].

### Majority of axonemal dyneins are missing or reduced in *pf23*

**Fig 2:**
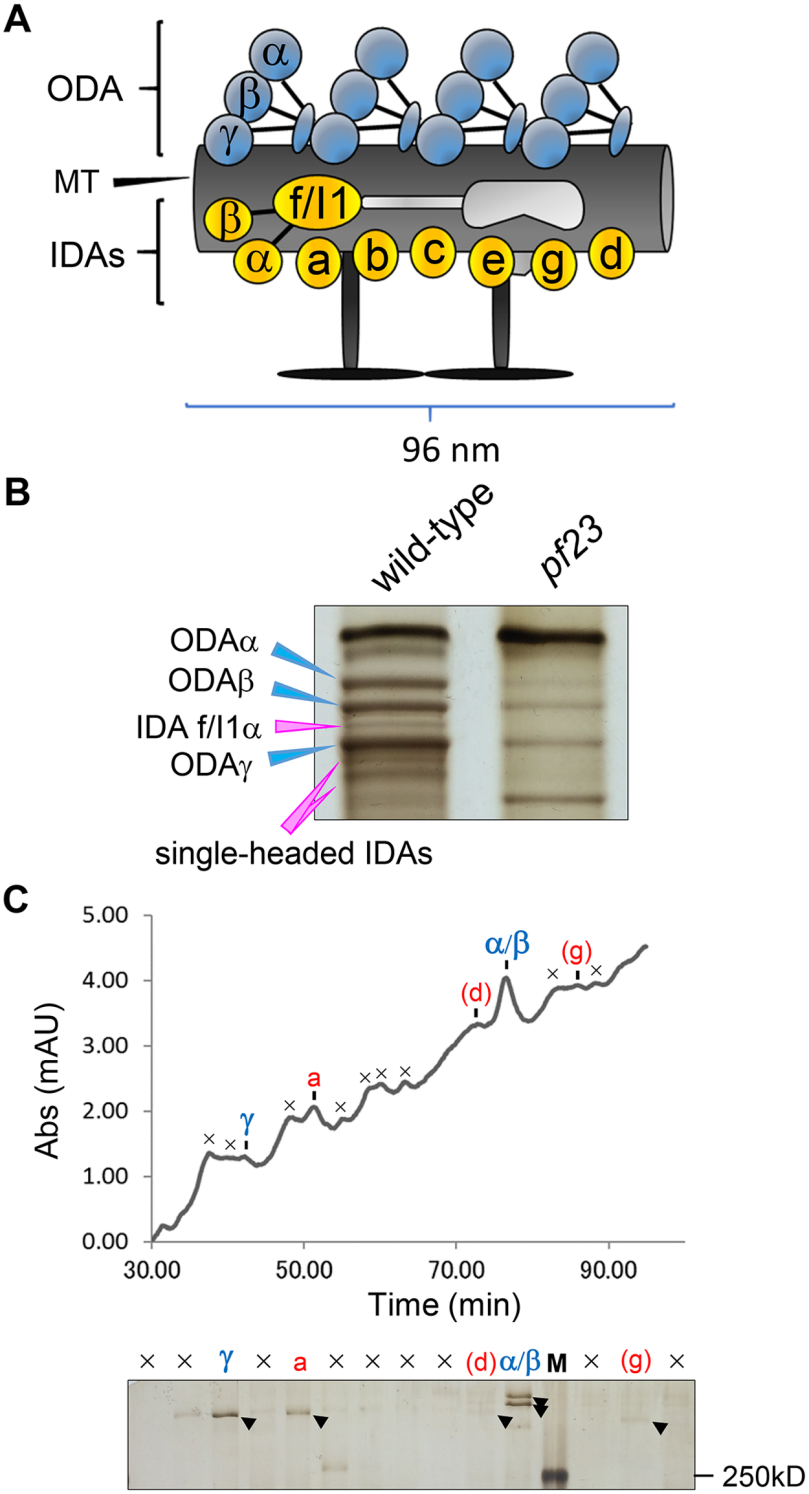
Ciliary dyneins, particularly IDAs, are greatly reduced in *pf23* axonemes. (A) Schematic drawing of a ciliary microtubule 96-nm repeat from *Chlamydomonas*. *Chlamydomonas* has one type of ODA consisting of three heavy chains (α, β and γ), and seven types of major IDAs (“a” to “g”). Among major IDAs, only IDA “f/I1” has two heavy chains (1α and 1β). In the proximal/distal part of the axonemes, some major IDAs are predicted to be replaced by three minor IDAs (“DHC3”, “DHC4” and “DHC11”). This figure is modified from Yamamoto et al., [57]. (B) Urea-PAGE of equal amount of ciliary axonemes from wild-type and *pf23*. Only the dynein heavy chain region of the gel is shown. Blue arrowheads indicate the three ODA heavy chains (α, β and γ), and pink arrowheads indicate the heavy chains of IDAs. Both the IDAs and ODA are greatly reduced in *pf23*. (C) Ciliary dyneins of *pf23* were separated using the Mono-Q column on the HPLC system [45] and the peak fractions were assessed by the urea-PAGE. In the urea gel, ODA “α/β”, ODA “γ”, and IDA “a” were detected as strong bands, and IDA “d” and IDA “g” were detected as weak bands. The symbol “×” in the chromatographic pattern and the urea gel indicates non-dynein peaks or peaks containing unidentified high-molecular protein(s). “M” in the urea gel is the marker lane (Also see the elution pattern of [79]).

Ciliary dyneins can be divided into the outer dynein arms (ODA) that reside on the outer circumference of the doublet microtubules and the inner dynein arms (IDA) that reside on the inner circumference (Fig 2A). *Chlamydomonas* has only one typeof ODA. There are seven major subspecies of IDAs termed “a” to “g” and three minor subspecies of IDAs called “DHC3”, “DHC4” and “DHC11” (Fig 2A) [8, 45, 46]. ODAs are particularly important for high ciliary beat frequency, while the IDAs are essential for ciliary waveform control [8, 41]. The three minor subspecies have been shown to replace the major subspecies in proximal part of the axoneme [46, 47], and are predicted to be important for bend initiation in cilia. Although thin-section electron micrographs and high-resolution 2D gel electrophoresis showed a reduction in dynein arm assembly in axonemes from *pf23* [37, 39, 40], the exact dynein species that failed to assemble was not determined. Furthermore, the original *pf23* was described as a mutant lacking inner dynein arm proteins and structures, but retaining most of the ODAs [37]. Subsequent analysis predicts that four IDAs, “a”, “c”, “d” and “f/I1” are missing in axonemes from *pf23* [48, 49].

**Fig 3:**
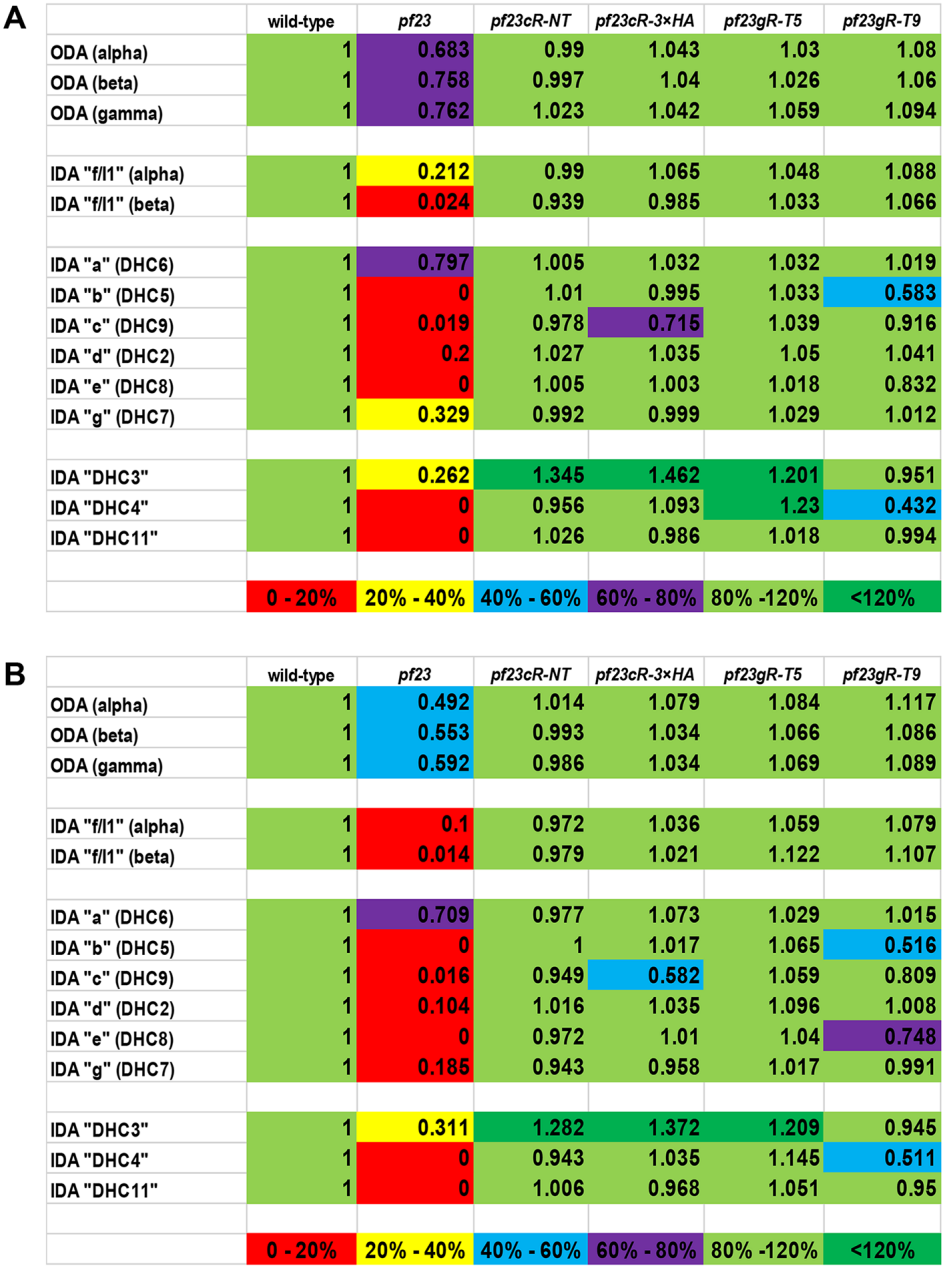
Spectral counting reveals failure of ciliary dynein assembly in *pf23* axonemes. (A, B) Ciliary dynein-containing regions of axonemal samples in the SDS-PAGE gels were cut from the gel, and the amount of each species of ciliary dynein estimated by spectral counting. The strains used were wild-type, *pf23*, *pf23cR-NT*, *pf23cR-3×HA*, *pf23gR-T5* and *pf23gR-T9*, and results were normalized using the wild-type spectral numbers. In (A), a summary of dynein heavy chain peptide fractions (unique peptides) is shown that verifies the reliability of the spectral counting analyses. In (B), a summary of unweighted peptide analyses used to semi-quantitatively estimate the amount of ciliary dynein heavy chains is shown. The average of two independent experiments is presented in the figures. The colors are an indication of the percentage of wild-type level of each dynein heavy chain: red 0–20%, yellow 20–40%, blue 40–60%, purple 60–80%, light green 80–120%, dark green < 120%, respectively. The *pf23* axonemes have greatly reduced amounts of major IDAs (“b”, “c”, “d”, “e”, “f/I1” and “g”), all below 20% of the wild-type level. In contrast, 70% of IDA “a” species is found in *pf23* axonemes. The minor IDAs, “DHC3”, “DHC4” and “DHC11”, are also greatly reduced in *pf23* axonemes. The ODA heavy chains are reduced about 50% compared to wild-type axonemes. In *pf23* rescued strains, most of ciliary dyneins are recovered, but major IDAs “b”, “e” and minor IDA “DHC4” remain reduced in *pf23gR-T9*, which expresses a short DYX1C1 fragment lacking the wild-type C-terminus.

To determine precisely which IDA subspecies are missing from *pf23* cilia, we used urea-PAGE [50, 51] to examine the dynein heavy chain composition. As described previously [37], urea-PAGE reveals that IDA bands are missing or reduced in density compared to wild-type (Fig 2B). To semi-quantitatively estimate the amounts of each dynein in cilia from *pf23*, we performed spectral counting of dynein heavy chain bands cut from the gel samples of wild-type and *pf23* as well as four of the *pf23* rescued strains described above (*pf23cR-NT*, *pf23cR-3×HA*, *pf23gR-T5* and *pf23gR-T9*) (S2 Table). Based on spectral counting, IDA subspecies “b”, “c”, “d”, “e”, “f/I1” and “g” are present at less than 20% of the wild-type level (Fig 3A and 3B), whereas inner dynein arm “a” is present at ~70% of the wild-type level (Fig 3B). We also found that ODAs have a modest defect in *pf23* as well as IDAs, ODAs are reduced to 50–60% of the wild-type level (Fig 3B). These results were also confirmed by HPLC chromatography using a Mono-Q column, which show clear reductions in all dynein peaks except inner dynein arm “a” (Fig 2C). In addition, we found that the minor IDA subspecies (“DHC3”, “DHC4” and “DHC11”) are missing or greatly reduced in *pf23* (Fig 3B). Taken together, the dynein defects in *pf23* are far more profound than previously described. Partial loss of the DYX domain in DYX1C1 results in an assembly defect for the majority of ciliary IDAs including the minor IDA subspecies and a fraction of the ODAs. Many of the mutations found in patients with PCD are premature stop codons (Y128X/W162X, V132X, and I195X). These stop codons may lead to mRNA decay. By the immunofluorescence microscopy with antibodies to ODA heavy chains, or to DNALI1 (dynein axonemal light intermediate chain 1) in IDAs, the dynein arms are not assembled in these patients [35]. These patients are likely to define the null phenotype, but the presence of the DYX1C1 protein was not monitored. At present, there is not a protein null for the *PF23* locus in *Chlamydomonas*. However, we would argue that the current *pf23* allele is not a complete null since some ODAs are assembled unlike in the PCD patients.

### Cryo-ET confirms the loss of dyneins in *pf23* axonemes

**Fig 4:**
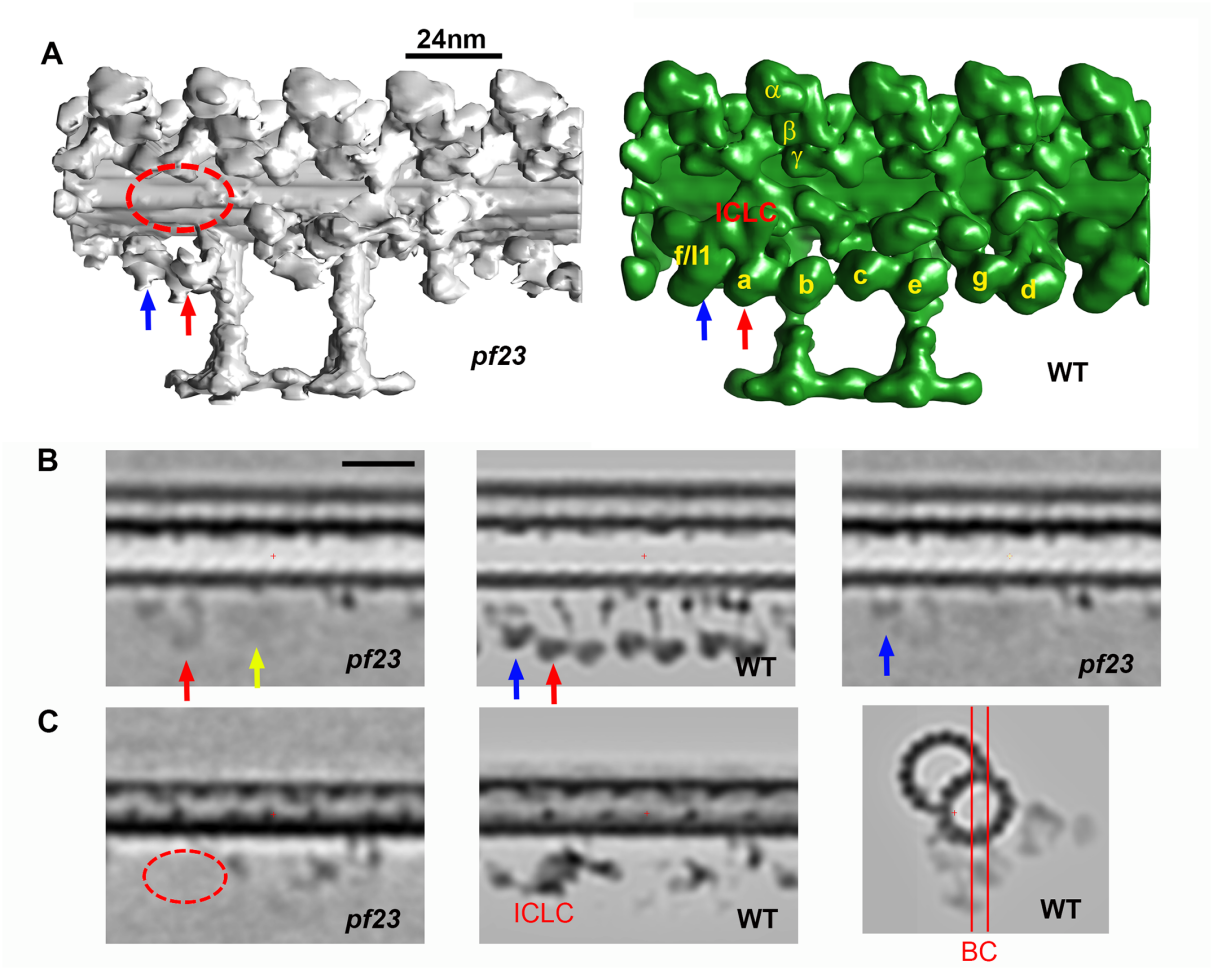
IDA defects in the *pf23* cilia visualized by Cryo-ET and image analysis. (A) Surface rendering representation of the averaged density maps from *pf23* (total average; left) and wild-type (EMD2131 [47]; right in green) cilia. Inner and outer arm dynein isoforms are indicated by yellow letters [47]. (B) Longitudinal sections from averaged cryo-ET density maps to compare *pf23* cilia, classified based on the area corresponding to the dynein “a” (left panel) and dynein “f/I1” (right) loci, respectively, and wild-type (center). Dynein “a” in wild-type and the density we interpret as dynein “a” in *pf23* are indicated by red arrows. There is density at the locus of dynein “f/I1”, but closer to the doublet microtubule (blue arrows in A and the right panel of B), which we interpret as non-dynein, possibly the tether defined by Heuser et al., [53]. Scale bar = 24 nm. (C) IC/LC complex of IDA “f/I1” is missing from *pf23* axoneme (encircled in the left panel). The central panel shows density of the IC/LC complex (indicated) sectioned at the same place as wild-type. In all the longitudinal sections and surface renderings, the proximal end of the axoneme is located at left and the distal end at right. The angles and positions of the sections in (B) and (C) are shown in the right panel. The analyses in Fig 4 were based on and further characterized/refined from [52] under the publisher’s permission (Elsevier).

To further assess defects of dynein structure in *pf23* axonemes, we performed cryo-electron tomography (cryo-ET) and subtomogram classification/average analysis of isolated axonemes. Sub-volumes containing a 96-nm periodic structure on the doublet microtubule were computationally extracted, aligned in 3D orientation, masked at dyneins of interest, classified and averaged (Fig 4 and [52]). The whole averaged map of *pf23* axonemes demonstrates a loss of most of IDAs, consistent with our biochemical analyses, which show a failure in assembly of most inner dynein arms (Fig 4A). A structure with an elongated and a globular density is seen at the position of dynein “a” (red arrow in Fig 4A, [52]), which suggests the presence of dynein “a”. To further characterize the structure located in the dynein “a” position (referred to as dynein “a”), and further assess occupancy of dyneins in the *pf23* axoneme, we employed a newly developed image classification technique [52]. In 71% of sub-tomograms, the dynein “a” structure appears more clearly in the sub-averages (Fig 4B: left), while in the remaining sub-tomograms dynein “a” is missing in sub-averages. Thus, consistent with the biochemical analysis in Fig 3A and 3B, cryo-ET reveals partial occupancy of the dynein “a” site in *pf23* axonemes.

With the exception of dynein “a”, cryo-ET analysis does not resolve other inner arm dyneins. There is a structure in *pf23* axonemes at the dynein “f/I1” position (blue arrows in Fig 4A and 4B). However, the structure does not show the morphology of a dynein. Features showing the head and the tail are not found and density corresponding to the IC/LC complex is missing (Fig 4A and 4C). The density at the dynein “f/I1” site is located at the position of the dynein “f/I1” tether, which is associated with the dynein “f/I1” motor domain: the tether is assembled in axonemes from other mutants missing f/I1 dynein [53]. Although the sites of dyneins “c” and “e” have diffuse intensity (yellow arrow in Fig 4B), we do not find any structure, before and after classification that look like a dynein. These densities may represent the IDA heavy chains that are not properly folded or represent density from other proteins. Similarly, no significant density is found at the location of the other inner dynein isoforms. Therefore, we conclude that only dynein “a” is found with partial occupancy in *pf23* axonemes. Given the large difference of intermediate/light chain compositions between the dynein species lacking in *pf23* (“b”, “c”, “d”, “e”, “f/I1”, “g”), these results strongly suggest that DYX1C1/PF23 plays an important role in assembly of ciliary IDA heavy chains. We also observed that, compared to wild-type axonemes, *pf23* axonemes show a tendency to be compressed during cryo preservation. This observation suggests the IDAs play a role in maintaining axonemal integrity.

**Fig 5:**
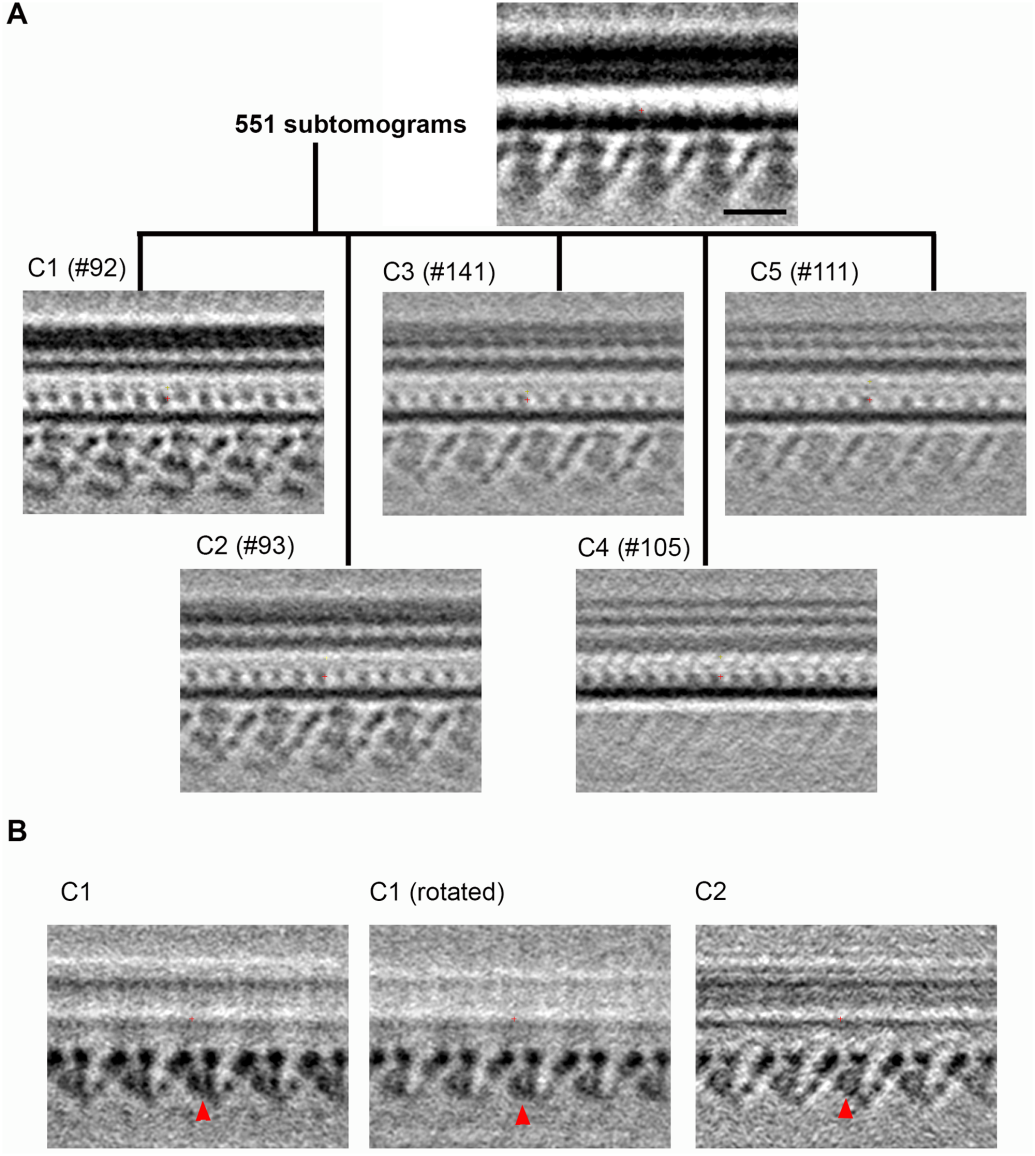
*pf23* also had slight structural defects in ODAs. (A) Classification of *pf23* ODAs based on the algorithm of Obbineni et al [52]. ODAs in *pf23* were classified into 5 classes. The total occupancy of ODAs in *pf23* was 81%, slightly more than our spectral counting results (Fig 3A and 3B). 9 doublets were not classified to any class. Scale bar = 24 nm. (B) Longitudinal sections which involve ODA β heavy chain heads (Left: C1 subclass, Center: C1 subclass, sectioned at the 7 degrees tilted angle with respect to the left panel. Right: C2 subclass). Resemblance of dynein heavy chain heads between the central and right panels suggest the β head domain in C1 subclass was tilted ~7 degree compared to that of C2 subclass. Dynein β heavy chain heads are indicated by red arrowheads. The proximal end of the axoneme is located at left and the distal end at right. The analyses in Fig 5 were based on and further characterized/refined from [52] under the publisher’s permission (Elsevier).

By cryo-ET observation and image classification, we can detect the decrease of ODAs, which is consistent with our biochemical analyses (Fig 5A). Subtomograms containing ODA were classified into five classes. One subclass (C4) shows no density of outer arm dynein heads. Since 19% of subtomograms were categorized in C4, we conclude that the total occupancy of ODAs in *pf23* cilia is about 81%, slightly more than our spectral counting results. Structural differences between other subclasses indicates heterogeneity of ODA in *pf23* axonemes. As partly described in Obbineni et al., [52], we noticed that one (β) of the three (α, β and γ) ODA heavy chains are tilted in some populations of ODAs in *pf23* axonemes. The observed ODA heterogeneity suggests DYX1C1/PF23 functions in the assembly of ODA heavy chains/heads (Fig 5B). In addition, partial failure in ODA assembly or heavy chain orientation may be a consequence of missing the outer-inner dynein (OID) linkers [47, 53–56]. Other obvious axonemal structures, such as radial spokes, central pair, N-DRC, and MIA complex [57] appear unaffected in the *pf23* axonemes.

### *Chlamydomonas* DYX1C1 functions in the cytoplasmic dynein preassembly pathway

**Fig 6:**
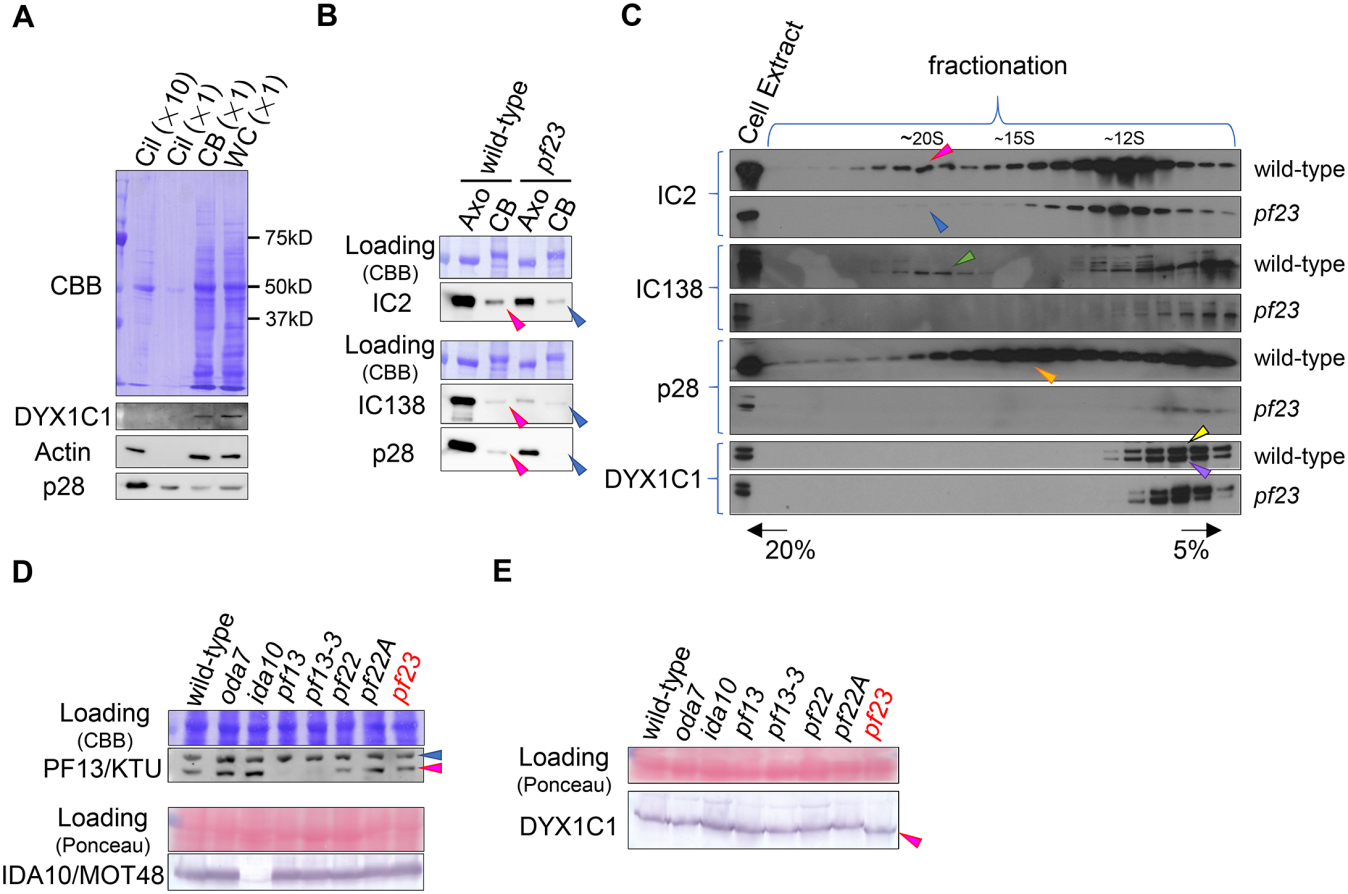
Ciliary dyneins are not preassembled in *pf23* cytoplasm. (A) *Chlamydomonas* DYX1C1 is located in the cell body. Wild-type whole cells (WC), deciliated cell bodies (CB), cilia (Cil) from the equivalent number of cell bodies, and a 10-fold excess of cilia were probed with anti-DYX1C1, anti-actin, and anti-p28 antibodies. While more than half of p28 and small amount of actin are present in ciliary IDAs, the signal of DYX1C1 was observed only in cell bodies and whole cell samples, indicating DYX1C1 functions in the cytoplasm. (B) The amounts of ciliary dynein subunits were greatly reduced both in axonemes and cell bodies of *pf23*. Total ~1 μg of axoneme (Axo) and cell bodies (CB) from wild-type and *pf23* were run on SDS-PAGE, and blotted with anti-IC2 (an intermediate chain of ODA), anti-IC138 (an intermediate chain of IDA “f/I1”) and anti-p28 (a light chain of IDAs “a”, “c”, “d”) antibodies. The amounts of these dynein subunits were greatly reduced in the cell bodies of *pf23* compared to wild-type (compare pink arrowheads to blue arrowheads), suggesting the stability of these subunits is reduced in *pf23* cytoplasmic extracts. (C) The cytoplasmic preassembly of ciliary dyneins is incomplete in *pf23* cytoplasm. Sucrose density centrifugation was performed on cytoplasmic extracts from wild-type and *pf23* to examine the preassembly of ODA and IDAs. Resultant fractions were probed with anti-IC2, anti-IC138, and anti-p28 antibodies. In wild-type cytoplasmic extracts, the pre-assembled ODA and IDAs sedimented as large complexes: ODA, ~20S, pink arrowhead; IDA “f/I1”, ~20S, green arrowhead; IDAs “a”, “c”, “d”, ~15S, orange arrowhead. In *pf23*, the pre-assembled IDA complexes (“a”, “c”, “d”, “f/I1”) were not observed, and only trace amount of preassembled ODA complexes were detectable (blue arrowhead). The fractions were also blotted using the anti-DYX1C1/PF23 antibody. PF23/DYX1C1 sedimented near the top of the sucrose density gradient fractions, suggesting the bindings between PF23/DYX1C1 and ciliary dyneins are transient or weak, and/or binding of only a small portion of DYX1C1 to ciliary dyneins is enough for the dynein pre-assembly process. A yellow arrowhead indicates the normal DYX1C1 band, while a purple arrowhead most likely indicates a band of slightly degraded DYX1C1 molecule. (D) Immunoblots of previously identified dynein preassembly factors (PF13/KTU/DNAAF2 and IDA10/MOT48) in whole cell samples from wild-type and various preassembly deficient mutants (*oda7*, *ida10*, *pf13*, *pf13-3*, *pf22*, *pf22A*, *pf23*) (S2 Table). The amount of the preassembly factors is variable from culture to culture, but relatively normal in *pf23* compared to wild-type. A pink arrowhead indicates the PF13/KTU bands, and a blue arrowhead above indicates non-specific bands. (E) Immunoblots of *Chlamydomonas* DYX1C1/PF23 on the whole cell samples from wild-type and various preassembly deficient mutants (*oda7*, *ida10*, *pf13*, *pf13-3*, *pf22*, *pf22A*, *pf23*). The amount of DYX1C1 is at wild-type levels in previously identified dynein-preassembly mutants. Note that *pf23* has a smaller DYX1C1 than other strains because of the mutation (pink arrowhead).

To ask if the cytoplasmic localization of DYX1C1 in mammals [35] is conserved in *Chlamydomonas*, we examined the cellular localization of DYX1C1 by immunoblots of *Chlamydomonas* cilia, cell bodies and whole cells using anti-DYX1C1 antibody (Fig 6A). DYX1C1 is found in cell bodies and whole cells, but not in cilia (Fig 6A). Although previous studies postulated that DYX1C1 could function in dynein assembly in the cytoplasm [35, 36], the exact state of ciliary dyneins in DYX1C1-deficient organisms remains unresolved. DYX1C1 could be required to maintain the stability of dynein subunits during assembly. To test this, we compared selected dynein subunits from the axoneme and cell body in wild-type and *pf23* cells by immunoblots (Fig 6B). As expected, IC2 (an intermediate chain of ODA), IC138 (an intermediate chain of IDA “f/I1”) and p28 (a light chain of IDAs “a”, “c”, “d”) are reduced in *pf23* axonemes (Fig 6B). Importantly, the relative amount of each protein is reduced in the cytoplasmic extracts (compare red and blue arrowheads, Fig 6B). Thus, the loss of part of the DYX domain appears to also be required for stability of axonemal dyneins before transport to and docking in the axoneme (Figs 1A and S1A).

To further test the idea that DYX1C1 is required for preassembly of ciliary dyneins in cytoplasm, we examined the preassembly state of ciliary dyneins in wild-type and *pf23* cytoplasmic extracts (Fig 6C). As previously reported for sucrose gradient fractionation of wild-type cytoplasmic extracts, IC2 sediments at ~20S and ~12S, IC138 sediments at ~20S, and p28 sediments at ~15S, respectively [11, 12]. In contrast, following sucrose density gradient centrifugation of *pf23* cytoplasmic extracts, the amount of preassembled ODA is greatly reduced compared to wild-type and the preassembly of IDAs is nearly undetectable (Fig 6C). These results indicate that *Chlamydomonas* DYX1C1 functions in cytoplasmic preassembly of ciliary dyneins.

### DYX1C1 may constitute a novel ciliary dynein preassembly complex

Based on a yeast two-hybrid experiment, Tarker et al [35] suggested a potential interaction between DYX1C1 and PF13/KTU/DNAAF2. Also, yeast two-hybrid and immunoprecipitation studies suggest interaction between DYX1C1 and PIH1D3, which is another preassembly factor responsible for X-linked primary ciliary dyskinesia [23, 24, 58]. To identify proteins that function with DYX1C1 in the preassembly/cytoplasmic stability of ciliary dyneins, we immunoprecipitated DYX1C1-containing complexes from the cytoplasm of the *pf23cR-3×HA* strain; an antibody to HA was used for precipitation from cytoplasmic extracts either with or without Bis(sulfosuccinimidyl)suberate (BS^3^)-crosslinking (see Materials and methods). From the immunoprecipitation experiments, we could not identify interactions between DYX1C1 and previously identified dynein preassembly factors reviewed in [16]. In addition, immunoblot analysis of whole cell extracts from wild-type and *pf23* do not reveal significant changes in the abundance of the previously identified dynein preassembly factors PF13/KTU/DNAAF2 and IDA10/MOT48 (Fig 6D). In *Chlamydomonas*, many double mutants that lack inner and outer dynein arms fail to assemble cilia. For example, the *pf9×oda4*, *ida3×oda2 or pf9×oda3* double mutants lack cilia [44, 59, 60]. We made double mutants of *pf23* with *pf13* (PF13/KTU/DNAAF2) or with *oda7* (ODA7/LRRC50/DNAAF1). The double mutants lacked cilia in both *pf23×pf13* and *pf23×oda7* strains. Furthermore, DYX1C1 levels are unaffected in the known preassembly deficient mutants, *oda7*, *ida10*, *pf13* and *pf22* (Fig 6E and S2 Table; reviewed in [16]). These results suggest that the stability and function of DYX1C1 in *Chlamydomonas* cells can be independent of the other preassembly factors. Given the differences in the dynein species missing in *pf23* compared to other preassembly mutants (*pf13*, *pf22*, *oda7* and *ida10*)(for the dynein defect(s) in each mutant see S2 Table), the DYX1C1/PF23 complex may have a unique molecular function in ciliary dynein pre-assembly. However, it is also possible that DYX1C1 functions both independently and cooperatively with previously identified preassembly factors for dynein assembly.

### Summary

We report that DYX1C1 is a ciliary dynein preassembly factor that is needed for dynein arm assembly; the DYX domain plays a major role in the preassembly of the inner dynein arms. We propose that DYX1C1 is needed for stability of ciliary dyneins both in cytoplasm and cilia. This action could be mediated by effects on protein folding as has been proposed for other preassembly factors, or by providing a scaffold for the association of various essential dynein components. Many of the previously identified preassembly factors have domains identified as playing roles in protein-protein interactions (e.g. TPR, HEAT, LRR), and may provide a unique staging area for the assembly of these large molecular complexes. Additional genetic, biochemical and structural analyses are required to determine the precise mechanistic role of DYX1C1 in dynein assembly.

## Materials and methods

### Strains and culture conditions

*Chlamydomonas reinhardtii* wild-type (137c) and the mutants that were used are listed in S2 Table. Mutants were purchased from *Chlamydomonas* Resource Center (University of Minnesota) or generously provided by Dr. Ritsu Kamiya (Chuo University). Cells were grown in TAP (Tris-Acetate-Phosphate) and/or SG (Sager and Granick) media under constant illumination.

### Preparation of cilia, axonemes, cell bodies and whole cell samples

Live *Chlamydomonas* cells were deciliated using the dibucaine method [61]. In order to prepare axonemes, detached cilia were collected by centrifugation, and demembranated with 0.2% IGEPAL or Nonidet P-40 in cold HMDEK (30 mM HEPES, 5 mM MgSO_4_, 1 mM DTT, 1 mM EGTA, and 50 mM potassium acetate, pH7.4) or HMDS (10 mM HEPES, 5 mM MgSO_4_, 1 mM DTT, 4% Sucrose, pH7.4) buffer. Membrane and matrix fractions were separated from the axonemes by centrifugation to yield a pure axoneme fraction. Extracts from cell body or whole cell samples were treated with methanol and chloroform to remove DNA/RNA fractions and washed with methanol several times until the resultant pellets became nearly white in color. In some experiments, to reduce possible protein degradation, isolated cell bodies or whole cells were directly added to SDS-PAGE sample buffer, mixed well, and heated at 95°C for 10 min.

### SDS-PAGE and immunoblots

SDS-PAGE and immunoblotting were performed with standard protocols described previously using 5 to 10% acrylamide gels [57]. SDS-PAGE gels were stained with CBB (Coomassie brilliant blue) or silver. For immunoblotting, samples separated by SDS-PAGE were transferred to a nitrocellulose or PVDF membrane, stained with CBB or Ponceau S if necessary, incubated with specific primary antibodies and subsequently HRP (horseradish peroxidase)-conjugated secondary antibodies. Immuno-reaction was detected using a TMB (3,3′,5,5′-tetramethylbenzidine peroxidase) substrate kit (Vector Laboratories), Pierce ECL immnoblotting substrate (Thermo Scientific) or ECL prime immunoblotting detection reagent (GE Healthcare). Primary antibodies used were as follows: anti-DYX1C1 CT299 (Rabbit: this study), anti-PF13/KTU (Rabbit: [17]), anti-IDA10/MOT48 (Rabbit: this study), anti-IC138 (Rabbit: [62]), anti-IC2 (Mouse: [63]), anti-Actin (Rabbit: [64]), anti-p28 (Rabbit: [59]), and anti-HA 3F10 (Rat: Roche Applied Science) or anti-HA Y11 (Rabbit: Santa Cruz). HRP-conjugated goat anti-rabbit or goat anti-mouse secondary antibodies were commercially purchased from Invitrogen. Anti-PF13/KTU antibody was a generous gift from Dr. David R. Mitchell (SUNY Upstate Medical University).

### Sucrose density gradient centrifugation

Sucrose density gradient centrifugation of *Chlamydomonas* cytoplasmic extracts was performed as described in [65]. Briefly, cells were broken by the glass beads method [66] and crude supernatant was obtained by centrifugation at 10,000 rpm for 10 min. The supernatant was clarified at 22,500 rpm for 2 hr using a Type 40 Beckman fixed-angle rotor, and the cytoplasmic extract was collected. The cytoplasmic extract was loaded on a 5–20% sucrose density gradient, centrifuged at 32,500 rpm for 16 hr using a Beckman SW41Ti rotor, and equal volume fractions were collected into Eppendorf tubes.

### Polyclonal antibody production

Anti-DYX1C1 antibody (CT299) was raised against maltose-binding protein fused to the 206-residue DYX1C1 sequence predicted by JGI *Chlamydomonas* genome version 4 (http://genome.jgi.doe.gov/Chlre4/Chlre4.home.html). This DYX1C1 antigen was synthesized by GenScript USA Inc. and corresponds to residues 1–142 and 324–350 of both the DYX1C1 sequence determined in this study and the full-length protein sequence predicted by Phytozome *Chlamydomonas* genome version 5.5 (https://phytozome.jgi.doe.gov/pz/portal.html#!info?alias=Org_Creinhardtii). Antibody was blot-purified [67] prior to use. An anti-MOT48 antibody was produced against the full-length MOT48 protein (see[18]) fused with the glutathione S-transferase protein located at the N-terminus. The MOT48 antiserum was blot-purified using the recombinant MOT48 protein.

### PCR for mapping

Crude DNA was obtained from about ~1×10^6^ cells from each progeny of matings and used for mapping PCR as previously described [68]. The matings were carried out between the S1D2 strain (CC-2290) and a *pf23* strain [69].

### Rescue of the *pf23* strain

Phenotypic rescue of *pf23* using a genomic DNA fragment containing the wild-type DYX1C1 sequence was performed as described previously [70]. Briefly, BAC29H10 was isolated from *Chlamydomonas* BAC library (bacterial artificial chromosome), and *pf23* cells were transformed with 29H10 by the glass bead method [71]. For screening of transformed *pf23* cells, the cells were grown in liquid TAP medium and swimming cells were isolated for further analyses.

Phenotypic rescue of *pf23* using wild-type *DYX1C1* cDNA and the pGenD vector was carried out as described previously [18, 72]. The wild-type *DYX1C1* cDNA was synthesized by GenScript Japan (http://www.genscript.jp/gene_synthesis.html), and inserted into the *Nde*I-*Eco*RI sites of the modified pGenD vector, which contains the *APHVIII* gene conferring paromomycin resistance to *Chlamydomonas*. For some experiments, the 3×HA epitope sequence was also inserted into the pGenD plasmid so that the exogenous *DYX1C1* gene would express a protein with the 3×HA tag at the C-terminus. The modified pGenD vector was transformed into *pf23* cells by the electroporation method [73]. Transformants were selected by growing the transformed *pf23* cells on TAP plates containing 10 μg/ml paromomycin. Swimming cells were then isolated for further study.

### Spectral counting of ciliary dynein heavy chains

For semi-quantitative estimation of amounts of axonemal dynein heavy chains, 15 μg axonemes were run on 8% acrylamide SDS-PAGE gels. Gel regions above 250 kDa containing all the axonemal dynein heavy chains were cut out and processed for spectral counting analyses using LC/MS/MS (liquid chromatography/MS/MS). The averages of two independent experiments are summarized in Fig 3.

### Identification of potential DYX1C1 interacting partners

To determine the potential interacting partners of DYX1C1, immunoprecipitation experiments were performed using an anti-HA antibody and cytoplasmic extracts from *pf23cR-3×HA* expressing 3×HA tagged exogenous DYX1C1. Immunoprecipitation was performed from extracts without chemical crosslinking or following BS^3^ (bis(sulfosuccinimidyl)suberate) crosslinking (0.5 mM). Cytoplasmic extracts of *pf23cR-3×HA* were obtained by sonicating cells that had been deciliated (by pH shock) 30 min to induce DYX1C1 expression. The cell extracts were clarified by centrifugation and the supernatants collected. Immunoprecipitation under non-crosslinked conditions was performed as described previously [57]. Cytoplasmic extracts were mixed with HA antibody-conjugated agarose beads (Anti-HA (3F10) affinity Matrix: Roche), incubated from several hours to overnight, and proteins precipitated with HA antibody-conjugated agarose beads run ~ 10 mm into an SDS-PAGE gel, stained with CBB, cut out from the gel and their identity determined by ESI/LC/MS/MS (electrospray ionization/liquid chromatography/MS/MS) analysis.

For immunoprecipitation under BS^3^-crosslinked conditions, cytoplasmic extracts were crosslinked with 0.5 mM BS^3^ for 30 min, and SDS-PAGE sample buffer containing 1% SDS added. Samples were then heated at 95°C for 10 min, diluted 10 times with 1% IGEPAL in TBS [74], and mixed with an HA-antibody conjugated agarose beads (Anti-HA (3F10) affinity Matrix: Roche). The mixture was incubated overnight and the proteins precipitated with the HA antibody-conjugated agarose beads processed as described above.

For experimental controls, untagged wild-type cytoplasmic extracts were processed in the same way as experimental samples, both under non-crosslinked and BS^3^-crosslinked conditions. The proteins identified in precipitates from *pf23cR-3×HA*, but not from wild-type, were considered as potential interacting partners of DYX1C1. The protein identification thresholds in Scaffold 4 software (http://www.proteomesoftware.com/products/scaffold/) were set as follows: Protein Threshold: 90%, Minimal Peptides Number: 2, Peptide Threshold: 80%.

### Cryo electron tomography

Purified cilia from *pf23* were mounted on lacey carbon grids (Plano, Germany) and plunged into liquid ethane at liquid nitrogen temperature using a hand-made manual plunger. Frozen grids were transferred to the JEM2200FS transmission electron microscope (JEOL, Japan) by a cryo-transfer system 626 (Gatan, USA). Electron micrographs were recorded at a nominal magnification of 20,000, accelerating voltage of 200 kV and total dose ~40e^-^/Å^2^, by in-column energy filtering (JEOL) and a CMOS detector F416 (TVIPS, Germany), using Serial EM [75]. Tomograms were reconstructed using IMOD. Subtomograms were extracted from axonemes located nearly parallel to the tilt axis of the goniometer of the microscope, aligned based on the 96-nm periodicity as described elsewhere [76], and classified using a missing wedge-free algorithm developed in part using axonemes from *pf23* [52]. For ODA classification, we set the number of subclasses to six. Since the sixth subclass contains only nine subtomograms which seem misaligned, we did not pursue further classification and show only five subaverages. 3D maps are presented using UCSF Chimera [77] and IMOD. The analyses of wild-type/*pf23* tomograms in Figs 4 and 5 were based on and further characterized/refined from [52] under the publisher’s permission (Elsevier). The density maps used in Figs 4 and 5 have been deposited at EMDataBank (http://www.emdatabank.org/) under the accession IDs EMD3779, EMD3786, and EMD3787.

### Other methods

Protein concentration of samples was measured by the Coomassie staining method [78]. Protein motifs and domains were analyzed using SMART (http://smart.embl-heidelberg.de/) or pfam (http://pfam.xfam.org/) searches. For sequence comparisons, data were aligned using ClustalW (http://www.genome.jp/tools/clustalw/) and the output was processed with BioEdit (http://www.mbio.ncsu.edu/bioedit/bioedit.html). Images were equally adjusted for contrast and/or brightness, if necessary. Figures were assembled using Photoshop (Adobe Systems), Microsoft Paint (Microsoft Windows), Illustrator (Adobe Systems), and/or Power Point (Microsoft Corporation).

## Supporting information

S1 Fig Domain structures of DYX1C1/PF23 and homologous proteins. (A) Domain structure was predicted using the SMART/Pfam analyses. DYX1C1 homologs have one CS domain (gray) at the N-terminal half, a coiled-coil region (green), and several TPR motifs (yellow) at the C-terminal half. The red dotted line in wild-type *Chlamydomonas* indicates the deficient region in *pf23*. The blue bars in human and *Chlamydomonas* structures represent the predicted positions of the DYX domain. The sequence NCBI accession numbers used for this comparison were as follows: Human, NP_570722; Mouse, NP_080590; Zebrafish, NP_991251; Trypanosoma, XP_829662. *Chlamydomonas* DYX1C1 sequences were determined in this study. (B) A phylogenetic tree of potential DYX1C1 homologs in eukaryotes. The alignment was created using Clustal W version 1.83 at DDBJ (http://www.ddbj.nig.ac.jp/index-j.html) at the default settings with neighbor-joining clustering. The tree was refined with NJplot (http://doua.prabi.fr/software/njplot). Another CS/PIH domain containing dynein pre-assembly factor, PIH1D3, was used as the outgroup. The sequence accession numbers used for tree construction were as follows: Human DYX1C1, NP_570722 (NCBI); Mouse DYX1C1, NP_080590 (NCBI); Zebrafish DYX1C1, NP_991251 (NCBI); Trypanosoma DYX1C1, XP_829662 (NCBI); Volvox DYX1C1, Vocar.0014s0240 (Phytozome Volvox v2.1); Tetrahymena DYX1C1, TTHERM_00540410 (TGD); Phytophthora DYX1C1, XP_009526226 (NCBI); *Chlamydomonas* DYX1C1, this study; *Chlamydomonas* TWI1, Cre07.g335800 (Phytozome *Chlamydomonas* v5.5); Human PIH1D3, NP_775765 (NCBI); Mouse PIH1D3, NP_808589 (NCBI); Zebrafish PIH1D3, NP_001002309 (NCBI); Trypanosoma PIH1D3, XP_821214 (NCBI).
(TIF)

S2 Fig Sequence comparison of DYX1C1/PF23 and homologous proteins. (A) DYX1C1/PF23 and its homologs in human, mouse and zebrafish were aligned using ClustalW. Red, black, and green bars represent the CS domain (a potential HSP90 binding module), the coiled-coil domain, and the TPR domain(s), respectively. Blue bar represents the DYX domain, which is defined in Chandrasekar et al [36]. The yellow dotted box indicates the 27-amino acid containing region deficient in *pf23*. (B) *Chlamydomonas* wild-type, mutated (*pf23*-type), and exogeneous DYX1C1 expressed in *pf23gR-T9* were aligned by ClustalW. The yellow dotted box indicates the 27-amino acid containing region deficient in *pf23*. The red bar represents a C-terminal region lacking in *pf23gR-T9*. The sequence NCBI accession numbers used for this comparison were as follows: Human, NP_570722; Mouse, NP_080590; Zebrafish, NP_991251. *Chlamydomonas* DYX1C1 sequences were determined in this study.
(TIF)

S1 Table Previously identified dynein pre-assembly factors present in *Chlamydomonas*. (DOCX)

S2 Table *Chlamydomonas* mutant strains used in this study. (DOCX)
