# Correction: *Chlamydomonas* DYX1C1/PF23 is essential for axonemal assembly and proper morphology of inner dynein arms

**DOI:** 10.1371/journal.pgen.1007063

**Published:** 2017-10-23

**Authors:** The PLOS Genetics Staff

The affiliation for the thirteenth author is incorrect. Susan K. Dutcher is not affiliated with #6, and only with #7 Department of Genetics, Washington University School of Medicine, St. Louis, Missouri, United States of America. The publisher apologizes for the error.
