# Supplementary figures and images for: Chlamydomonas DYX1C1/PF23 is essential for axonemal assembly and proper morphology of inner dynein arms
Source: PLoS Genet. 2017 Sep 11;13(9):e1006996. doi: 10.1371/journal.pgen.1006996 (PMC5608425; doi:10.1371/journal.pgen.1006996)

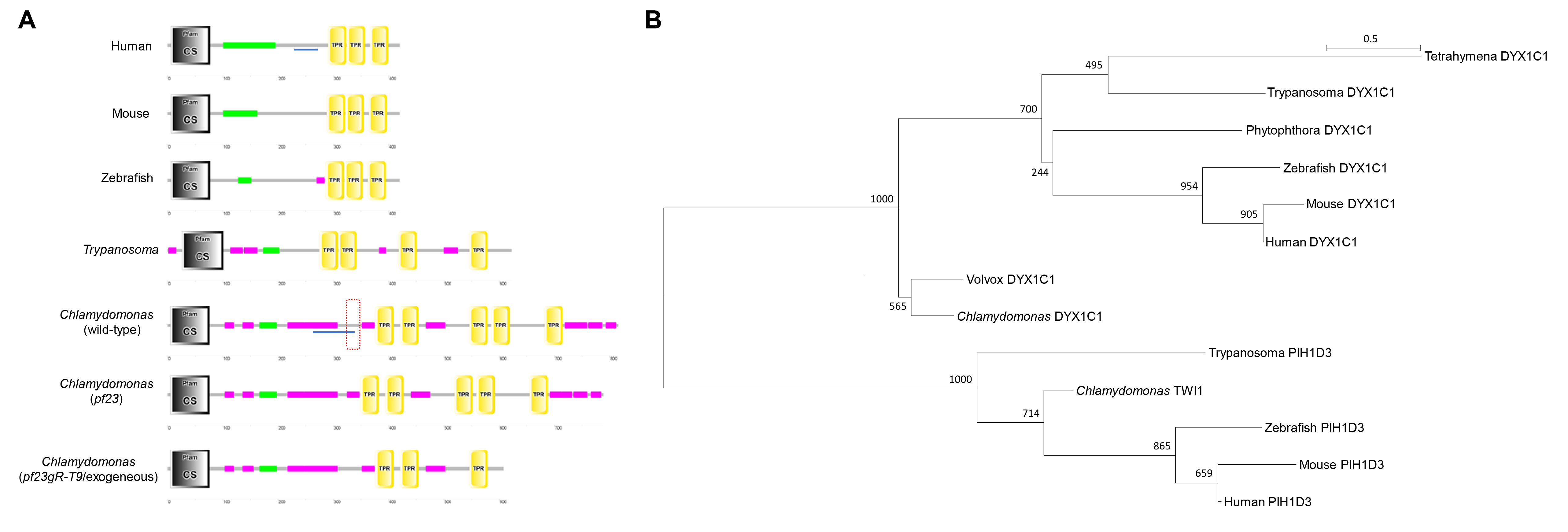

Supplement: S1 Fig — (A) Domain structure was predicted using the SMART/Pfam analyses. DYX1C1 homologs have one CS domain (gray) at the N-terminal half, a coiled-coil region (green), and several TPR motifs (yellow) at the C-terminal half. The red dotted line in wild-type Chlamydomonas indicates the deficient region in pf23. The blue bars in human and Chlamydomonas structures represent the predicted positions of the DYX domain. The sequence NCBI accession numbers used for this comparison were as follows: Human, NP_570722; Mouse, NP_080590; Zebrafish, NP_991251; Trypanosoma, XP_829662. Chlamydomonas DYX1C1 sequences were determined in this study. (B) A phylogenetic tree of potential DYX1C1 homologs in eukaryotes. The alignment was created using Clustal W version 1.83 at DDBJ (http://www.ddbj.nig.ac.jp/index-j.html) at the default settings with neighbor-joining clustering. The tree was refined with NJplot (http://doua.prabi.fr/software/njplot). Another CS/PIH domain containing dynein pre-assembly factor, PIH1D3, was used as the outgroup. The sequence accession numbers used for tree construction were as follows: Human DYX1C1, NP_570722 (NCBI); Mouse DYX1C1, NP_080590 (NCBI); Zebrafish DYX1C1, NP_991251 (NCBI); Trypanosoma DYX1C1, XP_829662 (NCBI); Volvox DYX1C1, Vocar.0014s0240 (Phytozome Volvox v2.1); Tetrahymena DYX1C1, TTHERM_00540410 (TGD); Phytophthora DYX1C1, XP_009526226 (NCBI); Chlamydomonas DYX1C1, this study; Chlamydomonas TWI1, Cre07.g335800 (Phytozome Chlamydomonas v5.5); Human PIH1D3, NP_775765 (NCBI); Mouse PIH1D3, NP_808589 (NCBI); Zebrafish PIH1D3, NP_001002309 (NCBI); Trypanosoma PIH1D3, XP_821214 (NCBI). (TIF) [file pgen.1006996.s001.tif]

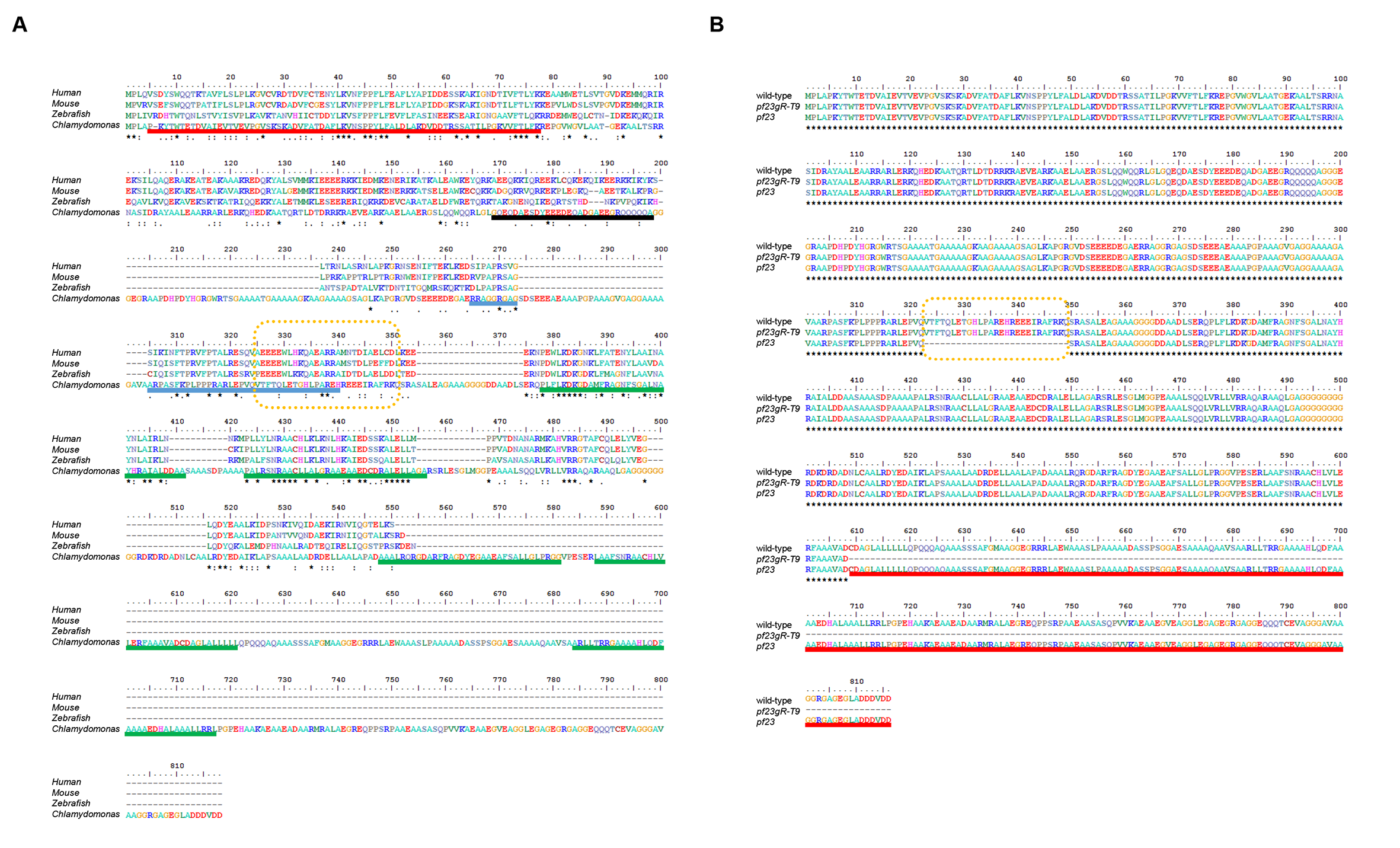

Supplement: S2 Fig — (A) DYX1C1/PF23 and its homologs in human, mouse and zebrafish were aligned using ClustalW. Red, black, and green bars represent the CS domain (a potential HSP90 binding module), the coiled-coil domain, and the TPR domain(s), respectively. Blue bar represents the DYX domain, which is defined in Chandrasekar et al [36]. The yellow dotted box indicates the 27-amino acid containing region deficient in pf23. (B) Chlamydomonas wild-type, mutated (pf23-type), and exogeneous DYX1C1 expressed in pf23gR-T9 were aligned by ClustalW. The yellow dotted box indicates the 27-amino acid containing region deficient in pf23. The red bar represents a C-terminal region lacking in pf23gR-T9. The sequence NCBI accession numbers used for this comparison were as follows: Human, NP_570722; Mouse, NP_080590; Zebrafish, NP_991251. Chlamydomonas DYX1C1 sequences were determined in this study. (TIF) [file pgen.1006996.s002.tif]
