# Supplementary material for: Chlamydomonas DYX1C1/PF23 is essential for axonemal assembly and proper morphology of inner dynein arms
Source: PLoS Genet. 2017 Sep 11;13(9):e1006996. doi: 10.1371/journal.pgen.1006996 (PMC5608425; doi:10.1371/journal.pgen.1006996)
Supplement: S1 Table — (DOCX) [file pgen.1006996.s003.docx]

**Supplemental Table 1. Previously Identified Dynein Pre-assembly Factors Present in *Chlamydomonas*.**

| **Gene/Protein Name** | **Phytozome (v5.5)** | **Dynein Defects**  **in *Chlamydomonas*** | **Domain Structure predicted by SMART/**  **pfam analyses** | **Mutant Name** | **Reference** |
| --- | --- | --- | --- | --- | --- |
| PF22/DAB1  DNAAF3 | Cre01.g001657 | ODA, some IDAs | DUF4470 | *pf22* | [1] |
| ODA7/LRRC50/  DAU1/DNAAF1 | Cre01.g029150 | ODA | LRR_9 | *oda7* | [2] |
| ODA8/MOT37/  DLU2 | Cre01.g043650 | ODA | None | *oda8* | [3] |
| SPAG1 | (Cre02.g084900)^a^ | N/A^b^ | TPR/RPAP3_C | N/A | [4] |
| ODA16/WDR69/  DAW1 | Cre04.g216902 | ODA | WD40 | *oda16* | [5, 6] |
| CCDC103 | Cre06.g253754/  Cre06.g253404 | N/A | RPAP3_C | N/A | [7-8] |
| PIH1D3/TWI1 | Cre07.g335800 | N/A | PIH1 | N/A | [9-11] |
| ODA5/DCC3 | Cre07.g342850 | ODA | Coiled-coil | *oda5* | [12, 13] |
| ZMYND10 | Cre08.g358751 | N/A | None | N/A | [14, 15] |
| ODA10 | Cre08.g361200 | ODA | Coiled-coil | *oda10* | [16] |
| HEATR2  DNAAF5 | Cre09.g395500 | ODA | None | N/A | [17, 18] |
| PF13/KTU/MOT45/DAP1/DNAAF2 | Cre09.g411450 | ODA, IDA “c” | PIH1/Coiled-coil | *pf13* | [1, 19] |
| Reptin/RUVBL2 | Cre10.g442700 | N/A | AAA | N/A | [20] |
| MOT48/DAP2 | Cre10.g467050 | Reduced amounts of  IDAs “b”, “c”, “d”, “e” | PIH1 | *ida10* | [21] |
| DYX1C1  DNAAF4 | Cre11.g467560 | Partial ODA, Almost complete loss of IDAs “b”, “c”, “d”, “e”, “f”, “g” (This study) | CS/Coiled-coil/TPR | *pf23* | [22, 23]  This Study |
| WDR92 | Cre16.g672600 | N/A | WD40 | N/A | [24] |
| LRRC6/Seahorse  /MOT47 | Cre17.g739850 | N/A | LRR/LRRcap/  Coiled-coil | N/A | [25, 26] |

^a^ parenthesis --- uncertain whether this is the real homologue of SPAG1

^b^ N/A --- not analyzed or not available

**References**

1. Mitchison HM, Schmidts M, Loges NT, Freshour J, Dritsoula A, Hirst RA, et al. Mutations in axonemal dynein assembly factor DNAAF3 cause primary ciliary dyskinesia. Nat Genet. 2012;44(4):381-9, S1-2. Epub 2012/03/06. doi: ng.1106 [pii]

10.1038/ng.1106. PubMed PMID: 22387996; PubMed Central PMCID: PMC3315610.

2. Freshour J, Yokoyama R, Mitchell DR. Chlamydomonas flagellar outer row dynein assembly protein ODA7 interacts with both outer row and I1 inner row dyneins. J Biol Chem. 2007;282(8):5404-12. PubMed PMID: 17194703.

3. Desai PB, Freshour JR, Mitchell DR. Chlamydomonas axonemal dynein assembly locus ODA8 encodes a conserved flagellar protein needed for cytoplasmic maturation of outer dynein arm complexes. Cytoskeleton (Hoboken). 2015;72(1):16-28. doi: 10.1002/cm.21206. PubMed PMID: 25558044; PubMed Central PMCID: PMCPMC4361367.

4. Knowles MR, Ostrowski LE, Loges NT, Hurd T, Leigh MW, Huang L, et al. Mutations in SPAG1 cause primary ciliary dyskinesia associated with defective outer and inner dynein arms. Am J Hum Genet. 2013;93(4):711-20. Epub 2013/09/24. doi: 10.1016/j.ajhg.2013.07.025. PubMed PMID: 24055112; PubMed Central PMCID: PMC3791252.

5. Ahmed NT, Mitchell DR. ODA16p, a Chlamydomonas flagellar protein needed for dynein assembly. Mol Biol Cell. 2005;16(10):5004-12. Epub 2005/08/12. doi: E05-07-0627 [pii]

10.1091/mbc.E05-07-0627. PubMed PMID: 16093345; PubMed Central PMCID: PMC1237099.

6. Ahmed NT, Gao C, Lucker BF, Cole DG, Mitchell DR. ODA16 aids axonemal outer row dynein assembly through an interaction with the intraflagellar transport machinery. J Cell Biol. 2008;183(2):313-22. PubMed PMID: 18852297.

7. Panizzi JR, Becker-Heck A, Castleman VH, Al-Mutairi DA, Liu Y, Loges NT, et al. CCDC103 mutations cause primary ciliary dyskinesia by disrupting assembly of ciliary dynein arms. Nat Genet. 2012;44(6):714-9. Epub 2012/05/15. doi: ng.2277 [pii]

10.1038/ng.2277. PubMed PMID: 22581229; PubMed Central PMCID: PMC3371652.

8. King SM, Patel-King RS. The oligomeric outer dynein arm assembly factor CCDC103 is tightly integrated within the ciliary axoneme and exhibits periodic binding to microtubules. J Biol Chem. 2015;290(12):7388-401. doi: 10.1074/jbc.M114.616425. PubMed PMID: 25572396; PubMed Central PMCID: PMCPMC4367249.

9. Dong F, Shinohara K, Botilde Y, Nabeshima R, Asai Y, Fukumoto A, et al. Pih1d3 is required for cytoplasmic preassembly of axonemal dynein in mouse sperm. J Cell Biol. 2014;204(2):203-13. doi: 10.1083/jcb.201304076. PubMed PMID: 24421334; PubMed Central PMCID: PMCPMC3897177.

10. Paff T, Loges NT, Aprea I, Wu K, Bakey Z, Haarman EG, et al. Mutations in PIH1D3 Cause X-Linked Primary Ciliary Dyskinesia with Outer and Inner Dynein Arm Defects. Am J Hum Genet. 2017;100(1):160-8. doi: 10.1016/j.ajhg.2016.11.019. PubMed PMID: 28041644; PubMed Central PMCID: PMCPMC5223094.

11. Olcese C, Patel MP, Shoemark A, Kiviluoto S, Legendre M, Williams HJ, et al. X-linked primary ciliary dyskinesia due to mutations in the cytoplasmic axonemal dynein assembly factor PIH1D3. Nat Commun. 2017;8:14279. doi: 10.1038/ncomms14279. PubMed PMID: 28176794; PubMed Central PMCID: PMCPMC5309803.

12. Wirschell M, Pazour G, Yoda A, Hirono M, Kamiya R, Witman GB. Oda5p, a novel axonemal protein required for assembly of the outer Dynein arm and an associated adenylate kinase. Mol Biol Cell. 2004;15(6):2729-41. PubMed PMID: 15064350.

13. Dean AB, Mitchell DR. Late steps in cytoplasmic maturation of assembly-competent axonemal outer arm dynein in Chlamydomonas require interaction of ODA5 and ODA10 in a complex. Mol Biol Cell. 2015;26(20):3596-605. doi: 10.1091/mbc.E15-05-0317. PubMed PMID: 26310446; PubMed Central PMCID: PMCPMC4603930.

14. Moore DJ, Onoufriadis A, Shoemark A, Simpson MA, zur Lage PI, de Castro SC, et al. Mutations in ZMYND10, a gene essential for proper axonemal assembly of inner and outer dynein arms in humans and flies, cause primary ciliary dyskinesia. Am J Hum Genet. 2013;93(2):346-56. Epub 2013/07/31. doi: 10.1016/j.ajhg.2013.07.009. PubMed PMID: 23891471; PubMed Central PMCID: PMC3738835.

15. Zariwala MA, Gee HY, Kurkowiak M, Al-Mutairi DA, Leigh MW, Hurd TW, et al. ZMYND10 is mutated in primary ciliary dyskinesia and interacts with LRRC6. Am J Hum Genet. 2013;93(2):336-45. Epub 2013/07/31. doi: 10.1016/j.ajhg.2013.06.007. PubMed PMID: 23891469; PubMed Central PMCID: PMC3738827.

16. Dean AB, Mitchell DR. Chlamydomonas ODA10 is a conserved axonemal protein that plays a unique role in outer dynein arm assembly. Mol Biol Cell. 2013;24(23):3689-96. Epub 2013/10/04. doi: 10.1091/mbc.E13-06-0310. PubMed PMID: 24088566; PubMed Central PMCID: PMC3842995.

17. Horani A, Druley TE, Zariwala MA, Patel AC, Levinson BT, Van Arendonk LG, et al. Whole-exome capture and sequencing identifies HEATR2 mutation as a cause of primary ciliary dyskinesia. Am J Hum Genet. 2012;91(4):685-93. Epub 2012/10/09. doi: S0002-9297(12)00434-X [pii]

10.1016/j.ajhg.2012.08.022. PubMed PMID: 23040496; PubMed Central PMCID: PMC3484505.

18. Diggle CP, Moore DJ, Mali G, zur Lage P, Ait-Lounis A, Schmidts M, et al. HEATR2 plays a conserved role in assembly of the ciliary motile apparatus. PLoS Genet. 2014;10(9):e1004577. doi: 10.1371/journal.pgen.1004577. PubMed PMID: 25232951; PubMed Central PMCID: PMCPMC4168999.

19. Omran H, Kobayashi D, Olbrich H, Tsukahara T, Loges N, Hagiwara H, et al. Ktu/PF13 is required for cytoplasmic pre-assembly of axonemal dyneins. Nature. 2008;456(7222):611-6.

20. Zhao L, Yuan S, Cao Y, Kallakuri S, Li Y, Kishimoto N, et al. Reptin/Ruvbl2 is a Lrrc6/Seahorse interactor essential for cilia motility. Proc Natl Acad Sci U S A. 2013;110(31):12697-702. doi: 10.1073/pnas.1300968110. PubMed PMID: 23858445; PubMed Central PMCID: PMCPMC3732945.

21. Yamamoto R, Hirono M, Kamiya R. Discrete PIH proteins function in the cytoplasmic preassembly of different subsets of axonemal dyneins. J Cell Biol. 2010;190(1):65-71. Epub 2010/07/07. doi: jcb.201002081 [pii]

10.1083/jcb.201002081. PubMed PMID: 20603327; PubMed Central PMCID: PMC2911668.

22. Huang B, Piperno G, Luck DJ. Paralyzed flagella mutants of *Chlamydomonas reinhardtii.* Defective for axonemal doublet microtubule arms. J Biol Chem. 1979;254(8):3091-9. PubMed PMID: 429335.

23. Tarkar A, Loges NT, Slagle CE, Francis R, Dougherty GW, Tamayo JV, et al. DYX1C1 is required for axonemal dynein assembly and ciliary motility. Nat Genet. 2013;45(9):995-1003. Epub 2013/07/23. doi: 10.1038/ng.2707. PubMed PMID: 23872636; PubMed Central PMCID: PMC4000444.

24. Patel-King RS, King SM. A prefoldin-associated WD-repeat protein (WDR92) is required for the correct architectural assembly of motile cilia. Mol Biol Cell. 2016;27(8):1204-9. doi: 10.1091/mbc.E16-01-0040. PubMed PMID: 26912790; PubMed Central PMCID: PMCPMC4831875.

25. Kott E, Duquesnoy P, Copin B, Legendre M, Dastot-Le Moal F, Montantin G, et al. Loss-of-function mutations in LRRC6, a gene essential for proper axonemal assembly of inner and outer dynein arms, cause primary ciliary dyskinesia. Am J Hum Genet. 2012;91(5):958-64. Epub 2012/11/06. doi: S0002-9297(12)00518-6 [pii]

10.1016/j.ajhg.2012.10.003. PubMed PMID: 23122589; PubMed Central PMCID: PMC3487148.

26. Horani A, Ferkol TW, Shoseyov D, Wasserman MG, Oren YS, Kerem B, et al. LRRC6 mutation causes primary ciliary dyskinesia with dynein arm defects. PLoS One. 2013;8(3):e59436. Epub 2013/03/26. doi: 10.1371/journal.pone.0059436. PubMed PMID: 23527195; PubMed Central PMCID: PMC3602302.
