# Supplementary material for: Chlamydomonas DYX1C1/PF23 is essential for axonemal assembly and proper morphology of inner dynein arms
Source: PLoS Genet. 2017 Sep 11;13(9):e1006996. doi: 10.1371/journal.pgen.1006996 (PMC5608425; doi:10.1371/journal.pgen.1006996)
Supplement: S2 Table — (DOCX) [file pgen.1006996.s004.docx]

**Supplemental Table 2. *Chlamydomonas* Mutant Strains Used in This Study.**

| **Mutant** | **Affected Protein** | **Defect/Description** | **Reference** |
| --- | --- | --- | --- |
| *ida10* | IDA10/MOT48 | Loss/reduced amount of  IDAs “b”, “c”, “d”, “e”  Modest reduction of ODA | [1] |
| *oda7*  (CC-2240) | ODA7/ LRRC50/DNAAF1 | Loss of ODA | [2, 3] |
| *pf13*  (CC-1030) | PF13/KTU/DNAAF2 | Loss of ODA  Loss of IDA “c” | [4, 5] |
| *pf13-3*  (CC-4185) | PF13/KTU/DNAAF2 | Loss of ODA  Loss of IDA “c” | [4, 5] |
| *pf22*  (CC-1382) | PF22/DNAAF3 | Loss of ODA and several IDAs (not exactly determined) | [5, 6] |
| *pf22A*  (CC-2493) | PF22/DNAAF3 | Loss of ODA and several IDAs (not exactly determined) | [5, 6] |
| *pf23*  Mating type +  (CC-3660) | DYX1C1/DNAAF4 | Loss of majority of IDAs  Loss of about half of ODA | This study, [5] |
| *pf23*  Mating type –  (CC-1383) | DYX1C1/DNAAF4 | Loss of majority of IDAs  Loss of about half of ODA | This study, [5] |
| *pf23gR-T1* | N/A | Rescued strain of *pf23* with a genomic fragment | This study |
| *pf23gR-T5* | N/A | Rescued strain of *pf23* with a genomic fragment | This study |
| *pf23gR-T9* | N/A | Rescued strain of *pf23* with a genomic fragment  Expression of a short DYX1C1 fragment | This study |
| *pf23gR-T14* | N/A | Rescued strain of *pf23* with a genomic fragment | This study |
| *pf23cR-3×HA* | N/A | Rescued strain of *pf23* with a cDNA fragment with a 3×HA tag | This study |
| *pf23cR-NT* | N/A | Rescued strain of *pf23* with a cDNA fragment without a tag | This study |
| S1D2  (CC-2290) | N/A | Having extensive sequence polymorphisms | [7] |

**References**

1. Yamamoto R, Hirono M, Kamiya R. Discrete PIH proteins function in the cytoplasmic preassembly of different subsets of axonemal dyneins. J Cell Biol. 2010;190(1):65-71. Epub 2010/07/07. doi: jcb.201002081 [pii]

10.1083/jcb.201002081. PubMed PMID: 20603327; PubMed Central PMCID: PMC2911668.

2. Kamiya R. Mutations at twelve independent loci result in absence of outer dynein arms in *Chlamydomonas reinhardtii*. J Cell Biol. 1988;107(6 Pt 1):2253-8. PubMed PMID: 2974040.

3. Freshour J, Yokoyama R, Mitchell DR. Chlamydomonas flagellar outer row dynein assembly protein ODA7 interacts with both outer row and I1 inner row dyneins. J Biol Chem. 2007;282(8):5404-12. PubMed PMID: 17194703.

4. Omran H, Kobayashi D, Olbrich H, Tsukahara T, Loges N, Hagiwara H, et al. Ktu/PF13 is required for cytoplasmic pre-assembly of axonemal dyneins. Nature. 2008;456(7222):611-6.

5. Huang B, Piperno G, Luck DJ. Paralyzed flagella mutants of *Chlamydomonas reinhardtii.* Defective for axonemal doublet microtubule arms. J Biol Chem. 1979;254(8):3091-9. PubMed PMID: 429335.

6. Mitchison HM, Schmidts M, Loges NT, Freshour J, Dritsoula A, Hirst RA, et al. Mutations in axonemal dynein assembly factor DNAAF3 cause primary ciliary dyskinesia. Nat Genet. 2012;44(4):381-9, S1-2. Epub 2012/03/06. doi: ng.1106 [pii]

10.1038/ng.1106. PubMed PMID: 22387996; PubMed Central PMCID: PMC3315610.

7. Gross CH, Ranum LP, Lefebvre PA. Extensive restriction fragment length polymorphisms in a new isolate of Chlamydomonas reinhardtii. Curr Genet. 1988;13(6):503-8. PubMed PMID: 2900078.
